# Biocontrol and Mycotoxin Mitigation: An Endophytic Fungus from Maize Exhibiting Dual Antagonism Against *Fusarium verticillioides* and Fumonisin Reduction

**DOI:** 10.3390/jof11060441

**Published:** 2025-06-11

**Authors:** Qianhui Li, Dongbeng Zhang, Dongyan Ye, Shuo Zhang, Qiurui Ma, Helong Bai, Fanlei Meng

**Affiliations:** 1 College of Chemistry, Changchun Normal University, Changchun 130032, China; qianyeahns@163.com (Q.L.); 17843305507@163.com (D.Z.); yedongyan0214@163.com (D.Y.); 15134342789@163.com (S.Z.); 13578927109@163.com (Q.M.); 2 Institute of Innovation Science and Technology, Changchun Normal University, Changchun 130032, China; 3 Institute of Agricultural Quality Standard and Testing Technology, Jilin Academy of Agricultural Sciences, Changchun 130033, China

**Keywords:** endophytic fungi, bioactive compounds, secondary metabolites, *Fusarium verticillioides*

## Abstract

*Fusarium verticillioides* is one of the pathogenic fungi causing maize ear rot, and its secreted fumonisins accumulated in plants pose significant threats to human health. To reduce the incidence of maize ear rot and fumonisin contamination, this study isolated numerous endophytic fungi from maize plants. Through inhibition zone and dual culture assays, an endophytic fungal strain, FJ284, demonstrating notable antifungal activity against *F. verticillioides* was screened. 18S rDNA gene sequencing was employed for fungal identification, and the sequences were deposited in NCBI GenBank. FJ284 was identified as *Penicillium oxalicum*. The ethyl acetate extract of *P. oxalicum* was analyzed using gas chromatography–mass spectrometry (GC-MS), revealing 52 compounds, including several secondary metabolites with documented anticancer, antimicrobial, and antioxidant activities. Furthermore, a spectroscopic method was developed to assess the inhibitory effect of strain FJ284 against *F. verticillioides*, showing maximum inhibition at 48 h. Finally, Ultra-High-Performance Liquid Chromatography–Mass Spectrometry (UHPLC-MS) analyses confirmed that FJ284 significantly inhibited three fumonisins (suppression rates > 50%), with efficacy ranked as fumonisin B3 (FB3) > fumonisin B2 (FB2) > fumonisin B1 (FB1).

## 1. Introduction

**Figure 1:**
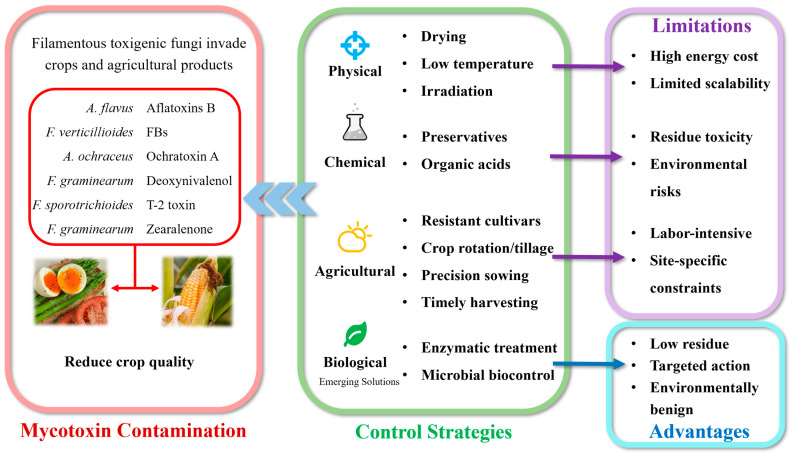
Contamination, challenges, and sustainable mitigation of mycotoxins in crops.

*Fusarium verticillioides* (*F. verticillioides*), a pathogenic fungus of maize, not only causes ear rot and root rot diseases in maize but also secretes the toxic mycotoxin fumonisin through its secondary metabolism. Among the fumonisins, FB1 is the most prevalent mycotoxin in both small-scale and commercial samples, exhibiting 100% and 98.6% contamination rates in small-scale and commercial samples, respectively [1]. Traditional measures for controlling the pathogens of maize ear rot primarily involve the application of fungicides. These fungicides have demonstrated significant efficacy in reducing pathogen contamination. For instance, Clodoaldo Fadani Andriolli et al. investigated the optimal timing for the application of azoxystrobin + cyproconazole and carbendazim as fungicides against maize pathogens [2]. The in vitro and field responses of 11 fungicides to a variety of major virus-producing fungi of maize, including *F. verticillioides*, were investigated [3]. The limitations of this conventional approach are twofold. First, fungicides generally need to be applied at specific growth stages of the plant, and their application may result in partial residue of the chemicals. Second, excessive use of fungicides can lead to a range of human health issues [4]. Figure 1 depicts prevalent mycotoxins and associated filamentous fungi with severe contamination risks alongside current mitigation methods and limitations of conventional approaches.

Endophytic fungi, residing asymptomatically within plant tissues while maintaining mutualistic relationships with their hosts, are pervasively distributed throughout diverse anatomical structures of various plant species [5]. The non-harmful nature of endophytic fungi towards their plant hosts facilitates their co-application with vegetation. Specifically, *Epichloë* species isolated from *Hordeum bogdanii*, a recognized salt-tolerant plant species, have been demonstrated to enhance plant tolerance to saline stress conditions. This symbiotic application of plants with endophytic fungi exhibits the potential to play a pivotal role in mitigating soil salinization challenges [6].

Endophytic fungi have garnered considerable scientific interest in contemporary research owing to their extraordinary biosynthetic capacity to generate a wide spectrum of secondary metabolites. These secondary metabolites not only mediate critical ecological dialogues between endophytic fungi and their plant hosts but also constitute an invaluable repository of bioactive molecules with multifaceted applications spanning pharmaceutical development [7], agricultural innovation [8], and industrial biotechnology [9]. Particularly noteworthy are fungal-derived metabolites demonstrating potent pharmacological activities, including anti-inflammatory, antimicrobial, and antioxidant properties, positioning these microorganisms as a frontier resource for next-generation drug discovery initiatives [10,11]. Walaa Kamel Mousa et al. isolated endophytic fungi with significant resistance to *F. verticillioides* from Finger Millet and identified the main antifungal compounds in these fungi [12]. Similarly, endophytic fungi from tomato plants have shown resistance to *Xanthomonas vesicatoria*, which causes bacterial spot disease in tomatoes [13].

Current biocontrol strategies against *F. verticillioides* predominantly focus on bacterial antagonism. Endophytic and epiphytic bacteria isolated from maize silk were screened for antifungal activity against *F. verticillioides* through in vitro and greenhouse assays. Four bacterial strains—*Achromobacter xylosoxidans* (ISD04), *Pseudomonas aeruginosa* (IPR45), and *Bacillus velezensis* (CT02 and IM14)—demonstrated significant in vitro antifungal activity [14]. In a parallel bacteriological approach, Phuong-Anh Nguyen et al. investigated the biocontrol efficacy of actinobacterial microbiota against *F. verticillioides*. Soil microbiota modification through metabolic intervention substantially suppressed fumonisin production, achieving 68.7% and 92.5% reductions in FB1 and FB2 levels, respectively [15]. Additionally, six bacterial isolates from *Euphorbia antiquorum* L. exhibited direct antagonistic activity against *F. verticillioides* in controlled experiments [16].

Fungi of the genus *Penicillium* serve as a source for a variety of antimicrobial and anti-inflammatory compounds. Van Cuong Pham et al. found several compounds from *Penicillium* sp. OM01 that exhibited antibacterial activity against Gram (+) bacteria, *Enterococcus faecalis*, *Staphylococcus aureus*, *Bacillus cereus*, and yeast *Candida albicans* [17]. Citromycin derivatives 1, 2, and 4, produced by *Penicillium* sp. TW131-64 and their corresponding enantiomers, showed significant antibacterial activity against *Helicobacter pylori* standard strains and multidrug-resistant strains and the effect was comparable to or even better than metronidazole [18].

Numerous studies have investigated *Penicillium* spp. against *Fusarium* spp. The fungal endophyte *Penicillium olsonii* ML37 can suppress *Fusarium graminearum* (the causal agent of *Fusarium* head blight in wheat) in vitro through a mechanism involving local defense activation in wheat spikes [19]. Han et al. discovered that *Penicillium caperatum* exhibits antagonistic effects against *Fusarium oxysporum*. Inoculation with MR-16 effectively suppressed disease lesions, demonstrating a control efficacy of 60.76% [20]. *Penicillium* sp. EU0013 also exhibits antagonistic activity against the *Fusarium* pathogen *Fusarium oxysporum*. In non-sterile soil, it significantly suppressed wilt symptoms in tomatoes (*Solanum lycopersicum* L.) and cabbages (*Brassica oleracea* L. var. *capitata*) [21]. Similarly, *Penicillium bilaiae* exhibits antagonistic effects against *Fusarium oxysporum*. Zhao et al. demonstrated that *Penicillium bilaiae* inhibited the mycelial growth of *Fusarium oxysporum* by 81.3% and overgrew the pathogen’s colonies in co-culture assays [22].

Prior research has exhibited that *P. oxalicum* is a potential source for producing bioactive withanolides, which possess a wide spectrum of therapeutic potentials [23]. Furthermore, *P. oxalicum* (QLhf-1) has been reported to have insecticidal properties against the aphid *Hyalopterus arundimis* Fabricius [24]. In a study by Dionisio G. Alvindia, *P. oxalicum* isolated from the surface of banana fruit was reported to have antagonistic activity towards *Lasiodiplodia theobromae* [25].

To date, research has mainly focused on exploring the antagonistic effects of fungi against *F. verticillioides* to prevent maize ear rot pathogenesis and reduce mycotoxin accumulation. This study, inspired by endophytic fungi as symbiotic microorganisms producing bioactive metabolites, capitalizes on the ecological advantage that these fungi are harmless or even beneficial to plant hosts. It aims to mitigate the proliferation of the harmful corn pathogen *F. verticillioides*, identify and characterize a fungus with potent antagonistic activity, and analyze its secondary metabolites using gas and liquid chromatography–mass spectrometry. Additionally, the study systematically evaluates the fungus’s ability to reduce fumonisin contamination.

## 2. Materials and Methods

### 2.1. Source of Endophytic Fungi

The endophytic fungi were isolated from fresh tissues of Fumin 228 maize plants (13 species collected from the Chaoyangpo area, 43.6211879 °N and 124.806907 °E) from Gongzhuling, Jilin Province (northeastern China). The collected samples were aseptically packaged in polythene bags under refrigerated conditions (4 °C). The endophytic fungi were isolated within a 24 h post-sampling window to ensure culturability.

The prepared plant samples included maize grains and maize stems. First, we placed the plant samples on a clean bench. Surface disinfection was performed as follows: the plant samples were soaked and disinfected with 75% ethanol for 30 s and, after the disinfection, the samples were rinsed thrice with sterile distilled water. This sterilization protocol was repeated in triplicate.

### 2.2. Isolation of Endophytic Fungi and Antifungal Experiment

Potato Dextrose Agar (PDA), peptone, glucose, KH_2_PO_4_·3H_2_O, MgSO_4_·7H_2_O, nalidixic acid, and agar were purchased from Shanghai Yuan Ye Co., Ltd. (Shanghai, China). The experiments utilized a PDA medium containing 4% Potato Dextrose Broth (PDB) and 2% agar, along with a modified Martin’s medium containing 1% peptone, 1% glucose, 0.1% KH_2_PO_4_·3H_2_O, 0.05% MgSO_4_·7H_2_O, 0.002% nalidixic acid, and 2% agar. Both media were autoclaved and poured into sterile Petri dishes while still liquid, then cooled to solidify before use. Endophytic fungi were isolated from maize grains and maize stems. For grains, samples were ground in a sterile mortar with 10 mL water, homogenized, and then a 100 μL suspension was spread on a PDA/modified Martin medium. For stems, surface-sterilized tissues were sectioned into 0.5 cm³ fragments and transferred to plates. Three fragments per plate were triangularly arranged in a 90 mm sized culture medium. All the plates were similarly sealed with parafilm. All inoculated media were transferred under aseptic conditions and incubated at 28 ± 1 °C for 3–5 days until well-developed colonies were observed. Colonies showing mixed morphologies were purified by streak-plating on PDA. Ultimately, a single purified strain was successfully isolated on PDA plates.

*F. verticillioides* was provided by the Institute of Agricultural Quality Standard and Testing Technology at the Jilin Academy of Agricultural Sciences. The following two types of antifungal experiments were conducted: a spread plate antagonistic assay and a dual culture antagonistic assay. For the spread plate antagonistic assay, small pieces of *F. verticillioides* were inoculated into PDB for 24 h at 28 ± 1 °C and at 180 rpm on a shaker. After completing the *F. verticillioides* culture, 250 µL of the supernatant was spread onto the new PDA plate, then a small piece of purified fungi (0.5 cm in diameter) was aseptically inverted and placed at the center of a PDA medium pre-coated with a *F. verticillioides* suspension. For the dual culture antagonistic assay, purified fungal cultures and *F. verticillioides* (mycelial plugs of 0.5 cm in diameter) were simultaneously inoculated at opposing ends of a PDA plate. We then incubated them for 3–5 days at 28 ± 1 °C to observe if there was a clear inhibition zone (with a diameter ≥ 10 mm) around the inoculated endophyte.

### 2.3. Sequence Analysis of P. oxalicum

The endophyte fungus was identified by Shanghai Sangon Biological Engineering Co., Ltd. This experiment utilized the Ezup Column Fungal Genomic DNA Extraction Kit to isolate genomic DNA from fungal strains. The extracted DNA served as a template for PCR amplification with primers NS1 (GTAGTCATATGCTTGTCTC) and NS6 (GCATCACAGACCTGTTATTGCCTC). The thermal cycling parameters included initial denaturation at 95 °C for 5 min, followed by 30 cycles at 94 °C for 30 s, 57 °C annealing for 30 s, and 72 °C extension for 90 s, with a final extension at 72 °C for 10 min. PCR products were analyzed by 1.5% agarose gel electrophoresis. The sequences were blasted against the NCBI databases. Combined morphological and sequence analyses determined the taxonomic classification at the order, family, or genus level.

### 2.4. Fermentation and Extraction of Secondary Metabolites from Endophytic Fungus

The endophytic fungi with antifungal ability (as outlined in Section 2.2 and identified in Section 2.3) were grown on PDA plates at 28 ± 1 °C for 4 days and then inoculated into 200 mL of PDB in Erlenmeyer flasks, followed by incubation under dark conditions at 28 ± 1 °C at 180 rpm on a shaker for 3 weeks. Following complete fermentation, the mycelium was separated from the fermentation broth. Equal amounts of ethyl acetate were added to the broth in the flask and allowed to stand for at least 24 h, then shaken thoroughly by hand and separated using a separating funnel. Concurrently, 100 mL of ethyl acetate was added to the mycelia in each flask, followed by ultrasonication for 30 min and 24 h incubation to extract secondary metabolites, with triplicate repetitions per flask. All ethyl acetate fractions from both the medium and mycelial extractions were pooled and concentrated using a rotary evaporator under reduced pressure, yielding crude solid-state fermentation products. The crude extract of the endophytic fungus was stored at 4 °C for further analysis.

### 2.5. Detection of Bioactive Compounds by a GC-MS Analysis

The ethyl acetate extract of FJ284 was subjected to GC-MS analysis to identify the bioactive compounds. The GC-MS analysis of the crude extracts was carried out using an Agilent 8890-7000D GC-MS system (Agilent Technologies, Santa Clara, CA, USA) with a TG-1701MS capillary column (30 m × 0.25 mm, 0.25 µm film thickness; Thermo Fisher Scientific, Waltham, MA, USA). The GC analytical conditions were systematically optimized based on the chromatographic parameters established by Kumar V et al. [26], with the following conditions: the oven temperature was initially set at 70 °C, increased to 90 °C at a rate of 2 °C/min, then raised to 180 °C at 4 °C/min and held for 10 min, followed by a ramp to 210 °C at 1 °C/min (held for 4 min), subsequently increased to 270 °C at 3.5 °C/min (held for 2 min), and finally elevated to 280 °C at 40 °C/min (held for 1 min). The sample was injected in splitless mode at a volume of 1.0 μL using nitrogen as the carrier gas. The mass spectral scan range was set at 30–600 *m*/*z* and 280 °C. The crude extract from Section 2.4 was dissolved in a 1:1 (*v*/*v*) acetone/*n*-hexane solvent mixture and subsequently analyzed by GC. The identification of the detected compounds was carried out by comparing them with the mass spectra from the NIST database.

### 2.6. Preliminary Screening Method for Mitigating Fumonisin-Producing Fungi

We aseptically prepared 100 mL of sterilized PDB and cut pre-cultured *F. verticillioides* (grown on PDA plates) and strain FJ284 into 5 mm² square fragments using a sterile scalpel. For the control group (DN01), we inoculated 1/4 of a *F. verticillioides* fragment into 100 mL PDB. For the experimental group (HN01), we inoculated 1/4 of a *F. verticillioides* fragment and 1/4 of an FJ284 fragment into a separate 100 mL PDB flask. Both flasks were incubated at 28 ± 1 °C with shaking (150 rpm) for 24 h. After 24 h, we combined 100 mL of the FJ284 culture with 100 mL of the *F. verticillioides* culture to form HN01, while DN01 was prepared by mixing 100 mL of the *F. verticillioides* culture with 100 mL fresh PDB. Both HN01 and DN01 were further incubated at 28 ± 1 °C with agitation. At daily intervals (1–7 days), we collected aliquots from each culture, filtered them through a 0.45 µm membrane to remove hyphae, and transferred 300 µL of the filtrate to a sterile 96-well plate. Measurements were conducted using a VersaMax microplate reader (Molecular Devices, San Jose, CA, USA). Optical density (OD) at 450 nm was measured using a microplate reader. The inhibition rate (IR) of *F. verticillioides* is calculated as

IR (%) = (A_1_ − A_2_)/(A_1_ − A_3_) × 100 (1)

where A_1_ is the absorbance of DN01, A_2_ is the absorbance of HN01, and A_3_ is the absorbance of PDB.

### 2.7. Ultra-High-Performance Liquid Chromatography–Mass Spectrometry (UHPLC-MS) Analysis of Fungal Co-Culture Extracts

We aseptically prepared 200 mL of sterilized PDB and cut pre-cultured *F. verticillioides* (grown on PDA plates) and strain FJ284 into 5 mm² square fragments using a sterile scalpel. For the control group (DN02), we inoculated 1/2 of a *F. verticillioides* fragment into 200 mL PDB. For the co-culture group (HN02), we inoculated 1/2 of a *F. verticillioides* fragment and 1/2 of an FJ284 fragment into a separate 200 mL PDB flask. Both flasks were incubated at 28 ± 1 °C with shaking (150 rpm) for 10 days. Post-incubation, we filtered the cultures through a 0.45 µm membrane, extracted the filtrate with methanol, and analyzed the methanol extracts via UHPLC-MS in triplicate.

The analysis was performed using a Nexera LC-30A Ultra-High-Performance Liquid Chromatography (UHPLC) system (Shimadzu Corporation, Kyoto, Japan) coupled with a QTRAP 5500 hybrid triple quadrupole linear ion trap mass spectrometer (AB Sciex, Framingham, MA, USA). The UHPLC-MS analysis was performed using a C18 column (2.1 × 100 mm; 1.7 µm particle size) maintained at 40 °C, with a flow rate of 0.3 mL/min and an injection volume of 3 µL. The mobile phase consisted of 0.1% formic acid with 5 mM ammonium formate in water (Phase A) and methanol (Phase B). The gradient program initiated at 30% B, increased linearly to 70% B over 2.3 min, held for 1.7 min, rapidly ramped to 100% B in 0.2 min, maintained for 1.8 min, returned to 30% B at 6.1 min, and re-equilibrated for 1.9 min. The detection employed electrospray ionization (ESI) in positive ion mode with a capillary voltage of 5.5 kV, using multiple reaction monitoring (MRM) for targeted metabolite quantification.

## 3. Results

### 3.1. Fungal Endophytes with Antibacterial Activity

Twenty-six samples were collected from maize plants in different fields at Gongzhuling for this study. From these 26 samples and the different parts of the maize plants, 80 fungi were isolated and purified (see Supplementary Materials Section S1).

**Figure 2:**
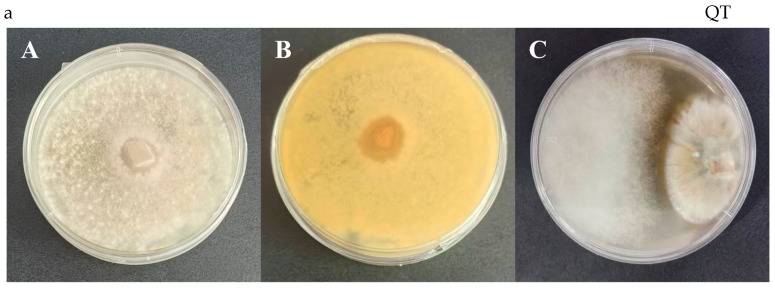
In vitro antagonistic activity of strain FJ284 against *F. verticillioides* in agar plate assays. (**A**) Front view of the agar plate; (**B**) back view of the agar plate; (**C**) interaction between *F. verticillioides* (L) and strain FJ284 (R).

**Figure 3:**
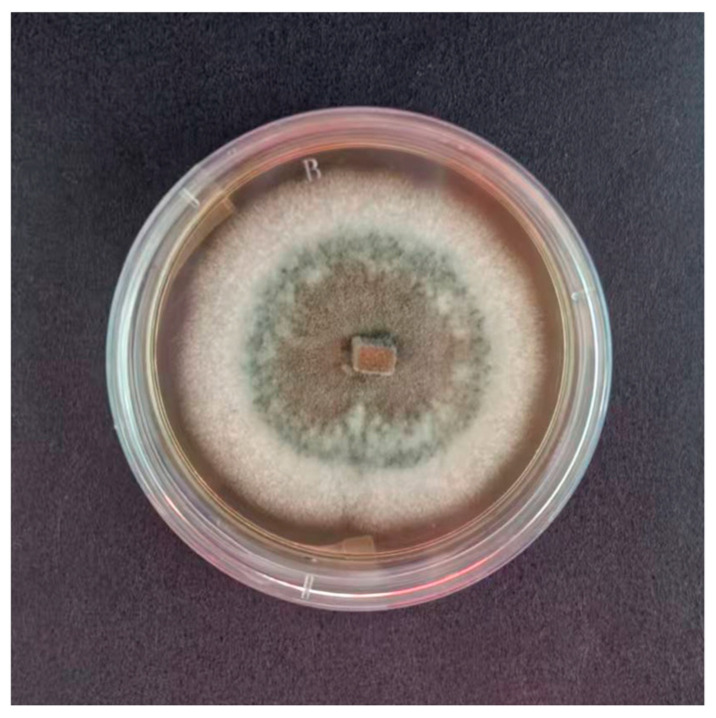
Strain FJ284 of *P. oxalicum*.

The purified fungus FJ284, which was isolated from maize stems, exhibited antifungal activity against *F. verticillioides*. FJ284 demonstrated antifungal activity by forming a distinct inhibition zone (Figure 2). In the spread plate antagonistic assay, strain FJ284 exhibited an inhibition zone diameter of 12.4 ± 1.5 mm (*n* = 3) against *F. verticillioides* and, in the dual culture antagonistic assay, the inhibition zone diameter of strain FJ284 against *F. verticillioides* was 4.6 ± 0.4 mm (*n* = 3). Through PCR amplification and 18s rRNA detection, the target band was 1270 bp in size, which matched the target strain of *P. oxalicum* (see Supplementary Materials Section S2). FJ284 was identified as *P. oxalicum* (Figure 3).

### 3.2. Analysis of Secondary Metabolites

**Figure 4:**
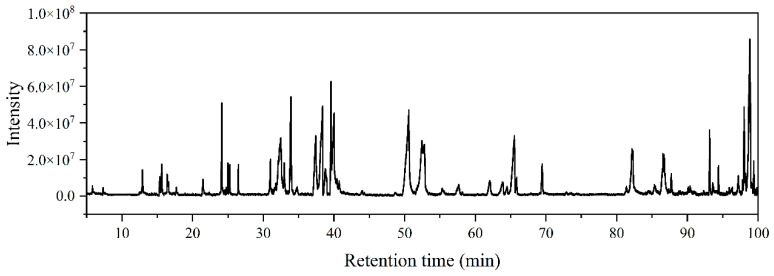
The chemical constituents in a crude extract of FJ284 identified using GC-MS.

The secondary metabolites of FJ284 were analyzed by gas chromatography attached to a mass spectrometer. Fifty-two compounds were found from FJ284, and the most abundant compounds were ergosterol (16.3%), pyrrolo[1,2-a]pyrazine-1,4-dione, hexahydro-3-(2-methylpropyl)- (10.89%), pyrrolo[1,2-a]pyrazine-1,4-dione, hexahydro-3-(phenylmethyl)- (9.57%), 9,12-Octadecadienoic acid (Z,Z)- (8.55%), and Cyclo(prolyl-tyrosyl) (6.66%) (Figure 4).

### 3.3. Results of Preliminary Screening and UHPLC-MS Evaluation

**Figure 5:**
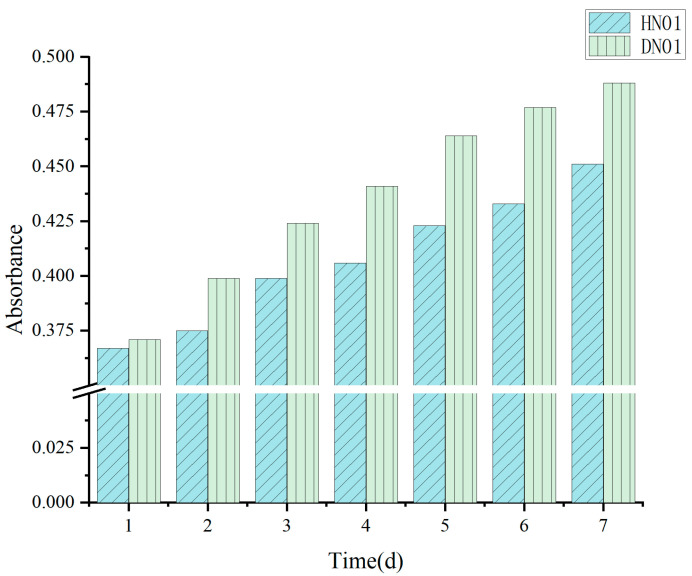
Changes in absorbance of HN01 and DN01 from day 1 to day 7.

As illustrated in Figure 5, *F. verticillioides* exhibited continuous fumonisin production during metabolic activity, with toxin accumulation increasing over time. Although the optical density (OD450) of the co-culture (HN01) displayed a gradual upward trend, its rate of increase was significantly attenuated compared with the monoculture control (DN01). This observation confirmed that the introduction of strain FJ284 substantially suppressed the OD450 of the mixed culture, indicative of the inhibitory effects on *F. verticillioides* growth or metabolic activity. The calculated inhibition rate (IR) peaked at 70.59% after 2 days of co-cultivation, aligning with the most pronounced reduction in OD450 at this timepoint.

**Figure 6:**
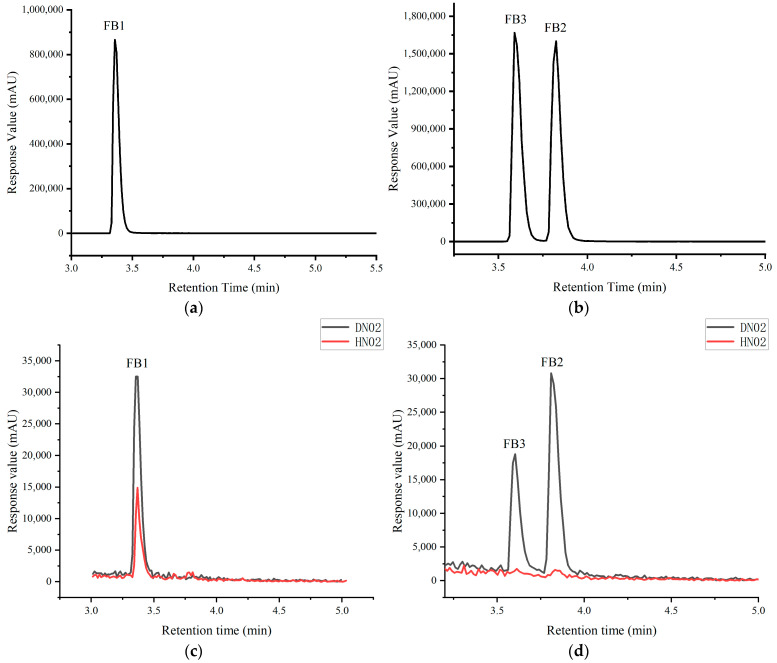
The standards of FB1 (**a**) and FB2 and FB3 (**b**) and the comparison of fumonisin reduction in DN02 and HN02 with FB1 (**c**) and FB2 and FB3 (**d**).

The UHPLC-MS analysis validated the efficacy of the preliminary screening method. Figure 6 distinctly reveals that in the co-culture system (HN02), all three fumonisin analogs (FB1, FB2, and FB3) exhibited reduced levels compared with the monoculture (DN02). Notably, FB3 was undetectable in HN02, suggesting near-complete suppression. FB2 was reduced by 95.752% and FB1 was reduced by 58.638%.

## 4. Discussion

From 26 maize samples (13 maize grains and 13 maize stems), 80 different fungal isolates were isolated and purified from different parts of maize samples. Through a spread plate antagonistic assay and a dual culture antagonistic assay, one isolate was preliminarily selected based on the efficiency of inhibiting *F. verticillioides* growth. In the spread plate antagonistic assay, the inhibition zone diameter of strain FJ284 against *F. verticillioides* was 12.4 ± 1.5 mm (*n* = 3), while in the dual culture antagonistic assay, the inhibition zone diameter of strain FJ284 against *F. verticillioides* was 4.6 ± 0.4 mm (*n* = 3). The isolate was identified as *P. oxalicum* through 18S rDNA gene sequencing. A dual culture antagonistic assay was also conducted in the study by Walaa Kamel Mousa et al. using isolated endophytic fungi, demonstrating inhibition zones with diameters ranging from 1 to 5 mm [12]. The dual culture antagonistic assay is an effective methodology for evaluating fungal–fungal antagonistic interactions, with our experimental findings exhibiting consistency with previously documented results in the literature. Building upon conventional confrontation assays, this study further implemented spread plate antifungal assays to more clearly and conclusively validate the antagonistic effect of strain FJ284 against *F. verticillioides*. The enhanced resolution of this methodological approach provides robust evidence for the biocontrol potential of the investigated fungal isolate.

The GC-MS analysis results of this study demonstrated the occurrence of fifty-two different compounds from the ethyl acetate extract of *P. oxalicum*. From the analysis, 19 compounds were identified to have biological activities such as antibacterial, antifungal, antioxidant, and anticancer activities (Table 1). A comparative GC-MS analysis of strain FJ284′s secondary metabolites cross-referenced with multiple literature sources revealed a diverse array of bioactive constituents exhibiting antibacterial, antifungal, and anticancer activities. It is hypothesized that the antagonistic efficacy of strain FJ284 against *F. verticillioides* could arise from the concerted action of multiple high-activity metabolites synthesized by this fungal isolate.

Different studies have reported that the same identical secondary metabolites can be produced by different fungi or even bacteria. For instance, pyrrolo[1,2-a]pyrazine-1,4-dione, hexahydro-3-(phenylmethyl)- was identified in the secondary metabolites of *Streptomyces* sp. HC14 and *Cladosporium cladosporioides* OP870014, which showed strong antimicrobial and anticancer activity [34,41]. Both pyrrolo[1,2-a]pyrazine-1,4-dione, hexahydro-3-(2-methylpropyl)- and pyrrolo[1,2-a]pyrazine-1,4-dione, hexahydro-3-(phenylmethyl)- are derivatives of pyrrolo[1,2-a]pyrazine-1,4-dione, having the same parent structure. Two compounds were identified via GC-MS analysis in the extracted volatile compounds of *Bacillus subtilis*, which showed biocontrol attributes to *Alternaria solani* [32]. Furthermore, a preceding study demonstrated the antimicrobial properties of pyrrolo[1,2-a]pyrazine-1,4-dione and cyclo(prolyl-tyrosyl) against *Candida albicans* [31].

The volatile compounds produced by *Achromobacter xylosoxidans* strain CTA8689 include the bioactive compound Benzene, 1,3-bis(1,1-dimethylethyl)-, which exhibits resistance against common bean root rot [27]. In the study of Aabid Manzoor Shah et al., actinomycin C2 showed better activity against *Staphylococcus aureus* ATCC 29213 and *Mycobacterium tuberculosis* strain H37Rv, with MIC values of 0.125 and 0.25 µg/mL [33]. The methanolic extract derived from *Olea europaea* leaves demonstrates a wide-ranging antibacterial efficacy, which includes the presence of 9,12-Octadecadienoic acid (Z, Z)- [53]. Recent studies have illustrated that ergosterol exhibits anticancer, antiproliferative, anti-inflammatory, and antimicrobial activities and also possesses the capacity to inhibit the synthesis and absorption of cholesterol within the human body [51,52]. Ergosterol not only has special physiological functions, but its derivatives also have great potential in drug development [50].

Fumonisin contamination in cereals, fruit, and agricultural commodities remains a global concern due to its documented health risks, driving sustained research focus on mycotoxin-detection methodologies. Matteo Ottoboni et al. evaluated the integration of electronic nose technology with lateral flow immunoassays for the rapid screening of aflatoxin/fumonisin occurrence and co-occurrence in maize samples [54].

In this investigation, to explore the regulatory effects of bioactive metabolites on suppressing *F. verticillioides*-mediated fumonisin biosynthesis, we developed an expeditious spectroscopic analytical method—a preliminary screening method—for the determination of fumonisin in fungal cultures. A preliminary screening analysis revealed that the absorbance values of HN01 demonstrated a significant reduction compared with DN01, indicating that the introduction of strain FJ284 could attenuate fumonisin biosynthesis in *F. verticillioides*.

Liquid chromatography–mass spectrometry (LC-MS) has been universally recognized as a benchmark analytical technique for mycotoxin detection due to its exceptional precision and high-throughput analytical capabilities. Juhee Park et al. demonstrated the utility of LC-MS in the simultaneous quantification of 12 mycotoxins, including fumonisins B1 and B2, in maize and derived products [55]. To enhance the data reliability of spectroscopic toxin profiling in the current study, cross-validation was implemented through UHPLC-MS, an advanced analytical platform characterized by a heightened sensitivity and chromatographic resolution. The UHPLC-MS analysis revealed that all three fumonisins exhibited reduced levels in HN02 compared with DN02. Notably, FB3 was undetectable in HN02, while FB2 was nearly undetectable. These findings indicate that the addition of FJ284 demonstrated optimal efficacy in suppressing *F. verticillioides*’ production of FB2 and FB3. A quantitative analysis based on peak area calculations showed a 95.752% reduction in FB2 and a 58.638% reduction in FB1.

The preliminary screening method demonstrated concordance with the UHPLC-MS detection results, further validating its reliability for the preliminary evaluation of fungal strains’ ability to suppress fumonisin production in *F. verticillioides*. Compared with UHPLC-MS detection, which requires sophisticated instrumentation and costly consumables, this screening method exhibits superior practicality for large-scale strain screening through simplified operational procedures, cost-effective instrumentation, and real-time detection capabilities.

Overall, strain FJ284 demonstrated multifaceted biotechnological potential in phytopathogen control. Although *P. oxalicum* has been previously documented to exhibit antibiosis against the banana pathogen *Lasiodiplodia theobromae* [25], our findings extend its antifungal spectrum to include the maize pathogen *F. verticillioides*. GC-MS metabolomic profiling revealed that FJ284 synthesized diverse volatile bioactive compounds, particularly antimicrobial and antioxidant substances, highlighting its dual applications in agricultural protection and pharmaceutical development. The UHPLC-MS analysis further demonstrated the antifungal activity of FJ284 to suppress fumonisin biosynthesis by the pathogen, suggesting an additional mycotoxin mitigation mechanism. These combined attributes position FJ284 as a promising resource for integrated crop disease management strategies. Future investigations should prioritize (i) the systematic isolation and structural characterization of secondary metabolites using advanced separation techniques and (ii) the development of conidia-based wettable powder formulations followed by phased field trials to evaluate their efficacy and ecological impact in mitigating fumonisin contamination.

## 5. Conclusions

In conclusion, this study successfully identified and characterized several bioactive compounds produced by fungi isolated from an antifungal experiment. Notably, *Penicillium oxalicum* demonstrated remarkable antifungal activity, highlighting its potential as a natural biocontrol agent against *F. verticillioides*. Upon analysis via GC-MS, the secondary metabolites were found to contain a variety of bioactive compounds, including volatile substances with notable antimicrobial properties.

Subsequently, UHPLC-MS was employed to evaluate the antimicrobial and detoxifying effects of FJ284. The results further confirmed that the addition of FJ284 exhibited certain antimicrobial properties against *F. verticillioides* and could effectively inhibit the secretion of toxic fumonisins to a certain extent. Additionally, the discovery of these compounds reinforces the hypothesis that endophytes of healthy plants are a promising and untapped source of highly bioactive substances, with significant potential for the development of novel antifungal agents and biocontrol strategies.

## Figures and Tables

**Table 1:** Analysis of bioactive components in the ethyl acetate extract of strain FJ284 by GC-MS.

No.	Name	RT/min	PCT	Bioactivity	References
1	Benzene, 1,3-bis(1,1-dimethylethyl)-	16.397	0.32%	Antimicrobial activity	[27]
2	dl-Mevalonic acid lactone	16.594	0.24%	Antifungal activity	[28]
3	Benzeneacetonitrile, 4-hydroxy-	24.131	2.75%	Inhibits cancer cells	[29]
4	4-Hydroxyphenylacetamide	32.376	1.72%	Antimicrobial activity	[30]
5	Cyclo(prolyl-tyrosyl)	34.009	6.66%		[31]
6	Pyrrolo[1,2-a]pyrazine-1,4-dione, hexahydro-3-(2-methylpropyl)-	38.478	10.89%	Antimicrobial activity and enzyme inhibition	[32]
7	Actinomycin C2	38.842	3.46%	Antimicrobial activity	[33]
8	Dibutyl phthalate	39.666	4.51%	Bioactive substance	[34]
9	*n*-Hexadecanoic acid	40.106	5.53%	Antiplasmodial	[35,36]
10	9,12-Octadecadienoic acid (Z,Z)-	50.671	8.55%	Anticancer activity and antibiotic-resistant	[37]
11	l-Leucyl-d-leucine	52.529	6.36%	Enzyme inhibition	[38]
12	Didemnin B	55.412	0.83%	Antiviral and anticancer activity	[39,40]
13	Pyrrolo[1,2-a]pyrazine-1,4-dione, hexahydro-3-(phenylmethyl)-	65.623	9.57%	Antimicrobial activity	[34,41]
14	Phenol, 2,2′-methylenebis[6-(1,1-dimethylethyl)-4-methyl-	69.532	2.17%	Anti-obesity activity and enzyme inhibition	[42,43]
15	Vernodalol	86.820	1.31%	Antioxidant and antitumor activity; enzyme inhibition	[44,45,46]
16	Ethyl iso-allocholate	90.460	0.30%	Antitumor and antimicrobial activity	[47,48]
17	7,8-Epoxylanostan-11-ol, 3-acetoxy-	94.907	0.21%	Bioactive substance	[49]
18	Ergosta-5,7,9(11),22-tetraen-3-ol, (3β,22E)-	98.120	3.43%	Anticancer, antiproliferative, anti-inflammatory, and antimicrobial activity	[50]
19	Ergosterol	98.936	16.30%		[51,52]

## Data Availability

The data presented in this study are available on request from the corresponding author. The data are not publicly available due to privacy and ethical restrictions.

## Supplementary Materials

The following supporting information can be downloaded at: https://www.mdpi.com/article/10.3390/jof11060441/s1, Section S1: Eighty fungi information; Section S2: Strain identification results.

## Abbreviations

The following abbreviations are used in this manuscript:

GC-MS	Gas Chromatography–Mass Spectrometry
UHPLC-MS	Ultra-High-Performance Liquid Chromatography–Mass Spectrometry
FB1	Fumonisin B1
FB2	Fumonisin B2
FB3	Fumonisin B3
PDA	Potato Dextrose Agar
PDB	Potato Dextrose Broth
