# Supplementary material for: Biocontrol and Mycotoxin Mitigation: An Endophytic Fungus from Maize Exhibiting Dual Antagonism Against Fusarium verticillioides and Fumonisin Reduction
Source: J Fungi (Basel). 2025 Jun 11;11(6):441. doi: 10.3390/jof11060441 (PMC12194516; doi:10.3390/jof11060441)
Supplement: Supplementary file 1 [file jof-11-00441-s001.zip › jof-3581686-supplementary.pdf]

Section S1. Eighty fungi information

NO.		Part of plant	Strain picture	NO.		Part of plant	Strain picture
1	FJ260	Maize grains	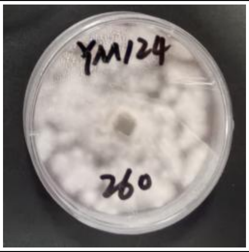	41	FJ384	Maize grains	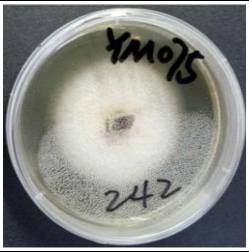
2	FJ261	Maize grains	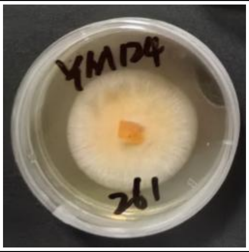	42	FJ385	Maize grains	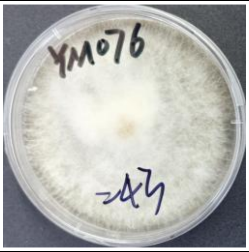
3	FJ262	Maize grains	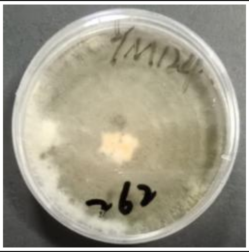	43	FJ386	Maize grains	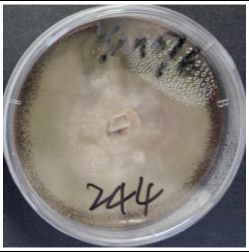
4	FJ264	Maize grains	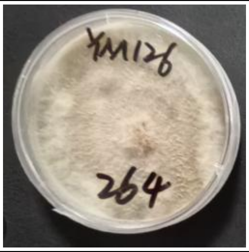	44	FJ387	Maize grains	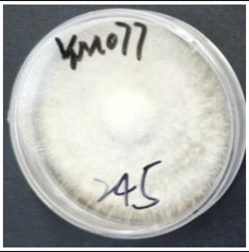
5	FJ265	Maize grains	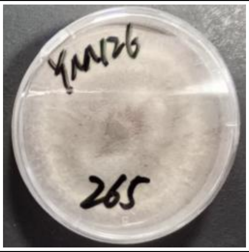	45	FJ388	Maize grains	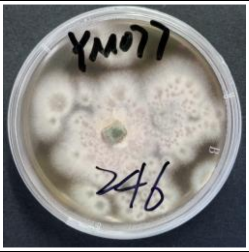
6	FJ266	Maize grains	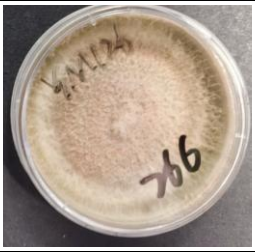	46	FJ389	Maize grains	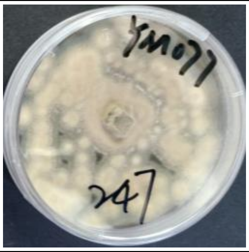

NO.		Part of plant	Strain picture	NO.		Part of plant	Strain picture
7	FJ267	Maize grains	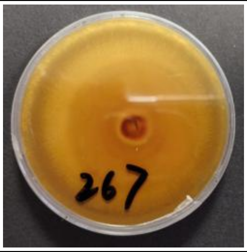	47	FJ390	Maize grains	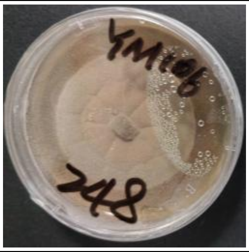
8	FJ268	Maize grains	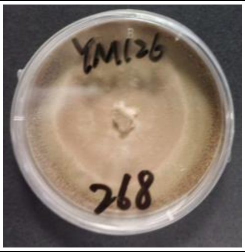	48	FJ392	Maize grains	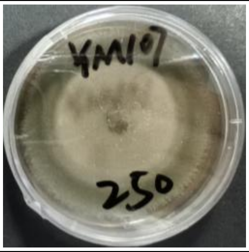
9	FJ269	Maize grains	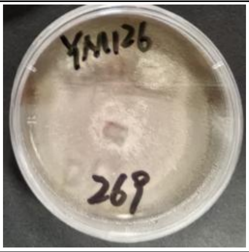	49	FJ393	Maize grains	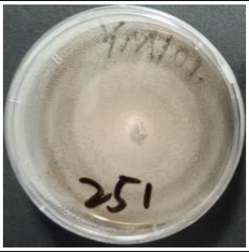
10	FJ270	Maize grains	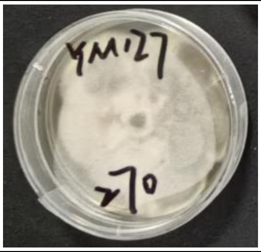	50	FJ394	Maize grains	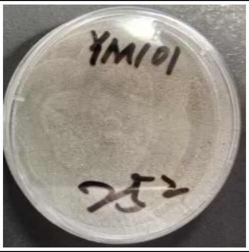
11	FJ271	Maize grains	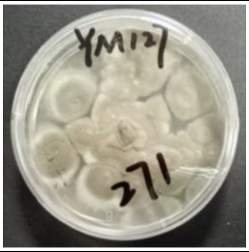	51	FJ395	Maize grains	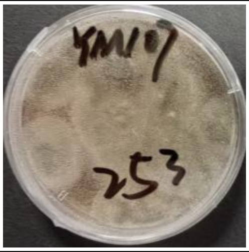
12	FJ343	Maize grains	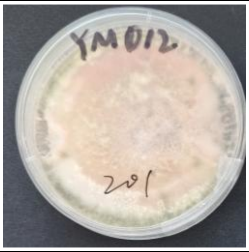	52	FJ398	Maize grains	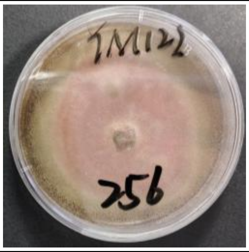
13	FJ344	Maize grains	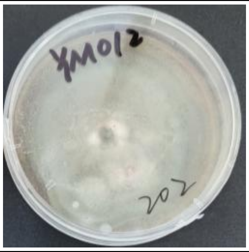	53	FJ399	Maize grains	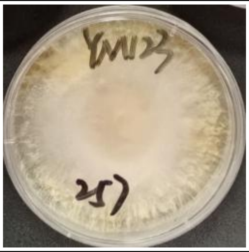

NO.		Part of plant	Strain picture	NO.		Part of plant	Strain picture
14	FJ345	Maize grains	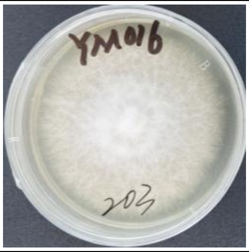	54	FJ400	Maize grains	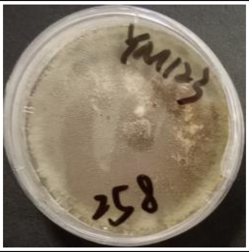
15	FJ346	Maize grains	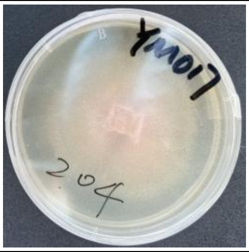	55	FJ272	Maize stem	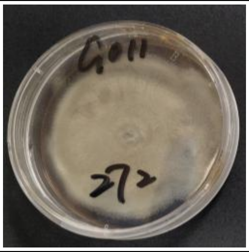
16	FJ347	Maize grains	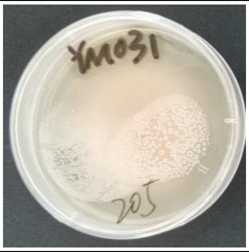	56	FJ273	Maize stem	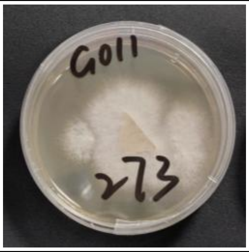
17	FJ348	Maize grains	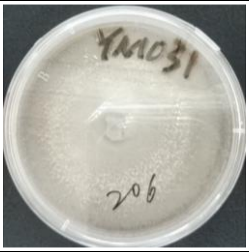	57	FJ274	Maize stem	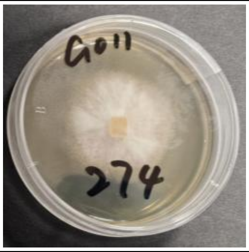
18	FJ349	Maize grains	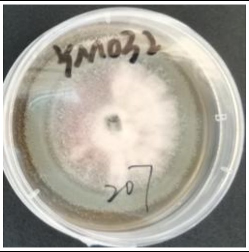	58	FJ275	Maize stem	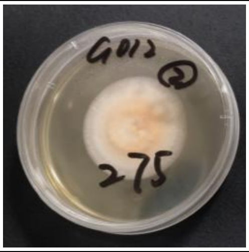
19	FJ358	Maize grains	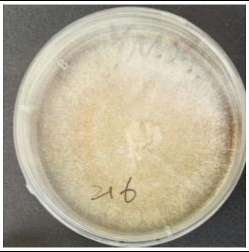	59	FJ276	Maize stem	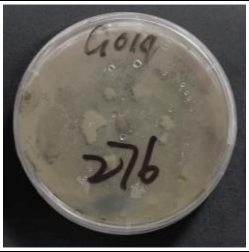
20	FJ359	Maize grains	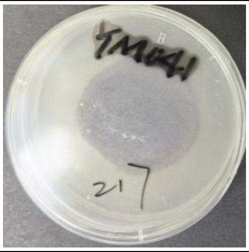	60	FJ277	Maize stem	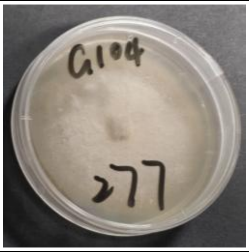

NO.		Part of plant	Strain picture	NO.		Part of plant	Strain picture
21	FJ361	Maize grains	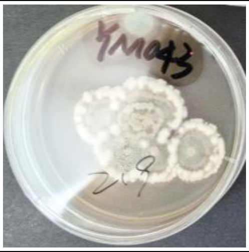	61	FJ278	Maize stem	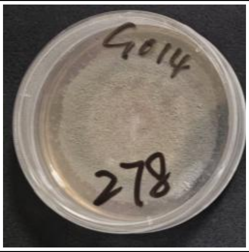
22	FJ362	Maize grains	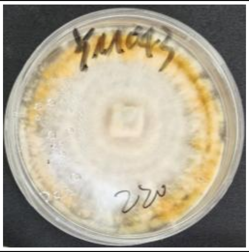	62	FJ279	Maize stem	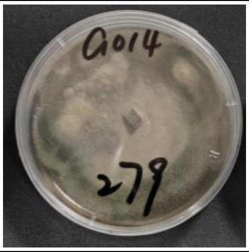
23	FJ363	Maize grains	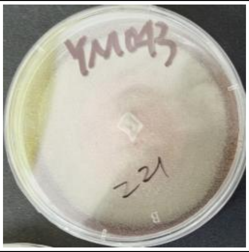	63	FJ280	Maize stem	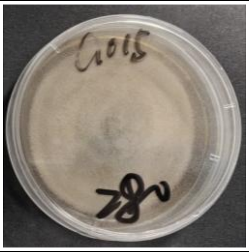
24	FJ364	Maize grains	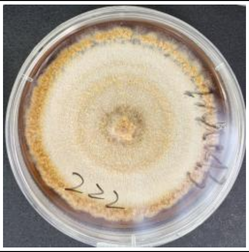	64	FJ282	Maize stem	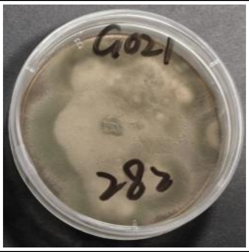
25	FJ365	Maize grains	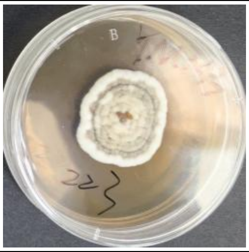	65	FJ283	Maize stem	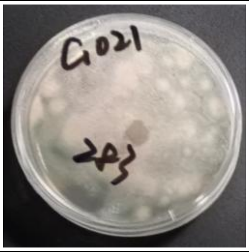
26	FJ366	Maize grains	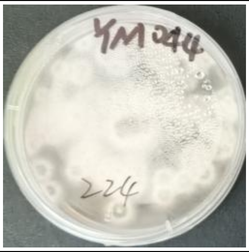	66	FJ284	Maize stem	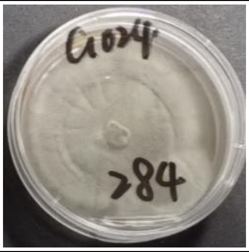
27	FJ367	Maize grains	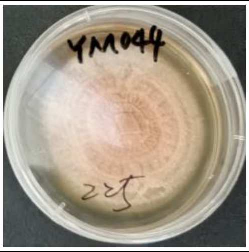	67	FJ285	Maize stem	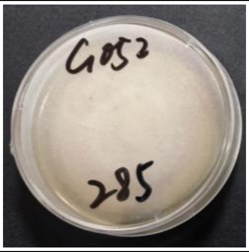

NO.		Part of plant	Strain picture	NO.		Part of plant	Strain picture
28	FJ369	Maize grains	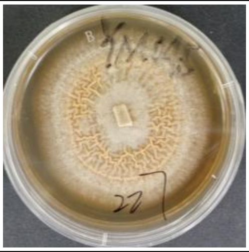	68	FJ286	Maize stem	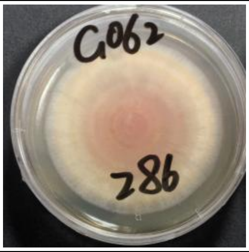
29	FJ370	Maize grains	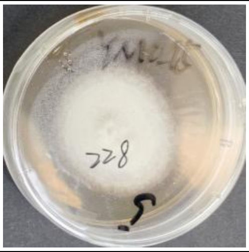	69	FJ287	Maize stem	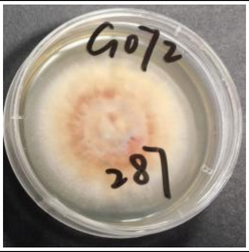
30	FJ371	Maize grains	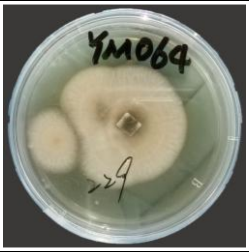	70	FJ288	Maize stem	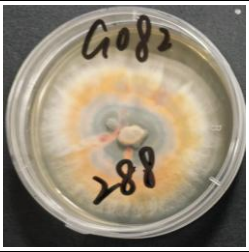
31	FJ372	Maize grains	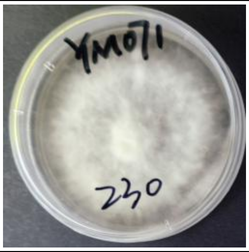	71	FJ289	Maize stem	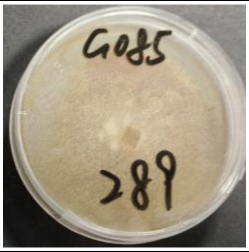
32	FJ374	Maize grains	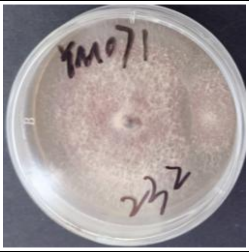	72	FJ290	Maize stem	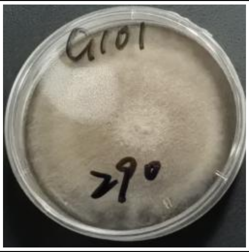
33	FJ376	Maize grains	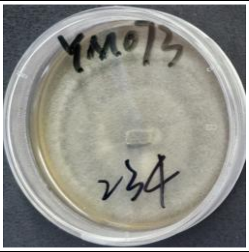	73	FJ291	Maize stem	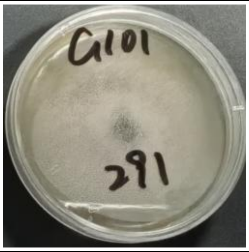
34	FJ377	Maize grains	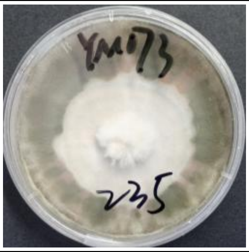	74	FJ292	Maize stem	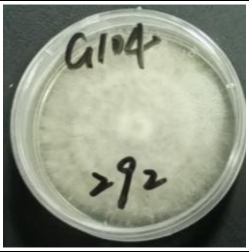

NO.		Part of plant	Strain picture	NO.		Part of plant	Strain picture
35	FJ378	Maize grains	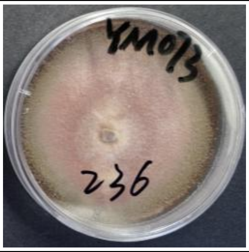	75	FJ293	Maize stem	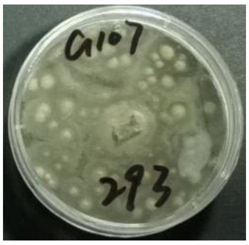
36	FJ379	Maize grains	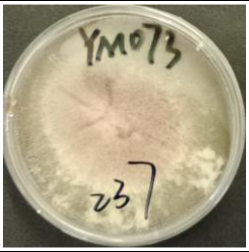	76	FJ294	Maize stem	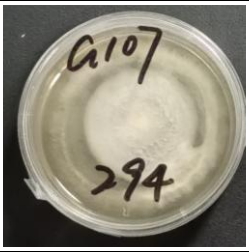
37	FJ380	Maize grains	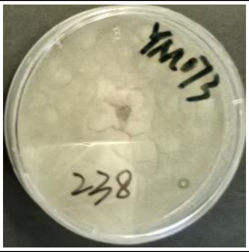	77	FJ295	Maize stem	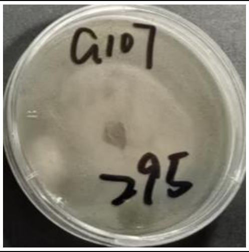
38	FJ381	Maize grains	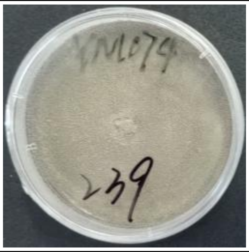	78	FJ296	Maize stem	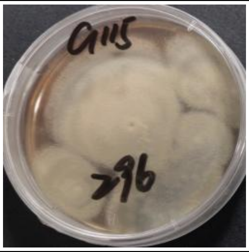
39	FJ382	Maize grains	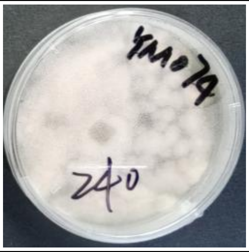	79	FJ297	Maize stem	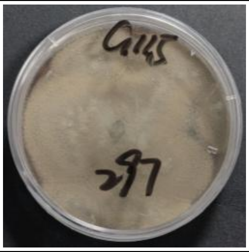
40	FJ383	Maize grains	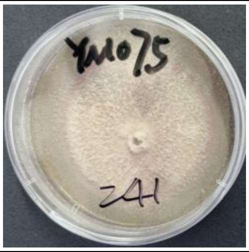	80	FJ298	Maize stem	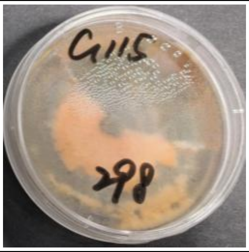

Section S2. Strain identification results

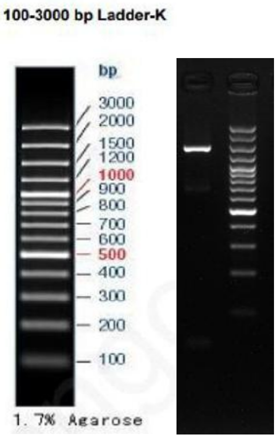

MK IS2465

IS2465 FJ284 1270bp

CTGCGAATGGCTCATTAAATCAGTTATCGTTTATTTGATAGTACCTTACTACATGGATACCTGTGGTAATT
CTAGAGCTAATACATGCTAAAAACCCCGACTTCAGGAAGGGGTGTATTTATTAGATAAAAAACCAACG
CCCTTCGGGGCTCCTTGGTGAATCATAATAACTTAACGAATCGCATGGCCTTGCGCCGGCGATGGTTCA
TTCAAATTTCTGCCCTATCAACTTTCGATGGTAGGATAGTGGCCTACCATGGTGGCAACGGGTAACGGG
GAATTAGGGTTCGATTCCGGAGAGGGAGCCTGAGAAACGGCTACCACATCCAAGGAAGGCAGCAGGC
GCGCAAATTACCCAATCCCGATACGGGGAGGTAGTGACAATAAATACTGATACGGGGCTCTTTTGGGTC
TCGTAATTGGAATGAGAACAATTTAAATCCCTTAACGAGGAACAATTGGAGGGCAAGTCTGGTGCCAG
CAGCCGCGGTAATTCCAGCTCCAATAGCGTATATTAAAGTTGTTGCAGTTAAAAAGCTCGTAGTTGAAC
CTTGGGTCTGGCTGGCCGGTCCGCCTCACCGCGAGTACTGTCCGGCTGGACCTTTCCTTCTGGGGAAC
CTCATGGCCTTCACTGGCTGTGGGGGGAACCAGGACCTTTTACTGTGAAAAAATTAGAGTGTTCAAAG
CAGGCCTTTGCTCGAATACATTAGCATGGAATAATAGAATAGGACGTGCGGTTCTATTTTGTTGGTTTCT
AGGACCGCCGTAATGATTAATAGGGATAGTCGGGGGCGTCAGTATTCAGCTGTCAGAGGTGAAATTCT
TGGATTTGCTGAAGACTAACTACTGCGAAAGCATTCGCCAAGGATGTTTTCATTAATCAGGGAACGAA
AGTTAGGGGATCGAAGACGATCAGATACCGTCGTAGTCTTAACCATAAACTATGCCGACTAGGGATCG
GACGGGATTCTATGATGACCCGTTCGGCACCTTACGAGAAATCAAAGTTTTTGGGTTCTGGGGGGAGT
ATGGTCGCAAGGCTGAAACTTAAAGAAATTGACGGAAGGGCACCACAAGGCGTGGAGCCTGCGGCTT
AATTTGACTCAACACGGGGAAACTCACCAGGTCCAGACAAAATAAGGATTGACAGATTGAGAGCTCT
TTCTTGATCTTTTGGATGGTGGTGCATGGCCGTTCTTAGTTGGTGGAGTGATTTGTCTGCTTAATTGCGA
TAACGAACGAGACCTCGGCCCTTAAATAGCCCGGTCCG

Description	Max Score	Total Score	Query Cover	E value	Per. Ident	Accession
Penicillium oxalicum strain TGQM01 small subunit ribosomal RNA gene, partial sequence	2338	2338	100%	0.0	99.92%	MK069498.1
Penicillium oxalicum strain SL2 small subunit ribosomal RNA gene, partial sequence	2338	2338	100%	0.0	99.92%	MG585101.1
Penicillium oxalicum clone EF036 small subunit ribosomal RNA gene, partial sequence	2338	2338	100%	0.0	99.92%	MG015950.1
Penicillium oxalicum strain FS12 small subunit ribosomal RNA gene, partial sequence	2338	2338	100%	0.0	99.92%	OR673586.1
Penicillium oxalicum strain 18S small subunit ribosomal RNA gene, partial sequence	2338	2338	100%	0.0	99.92%	OP538573.1
Penicillium limosum strain DHBS8 small subunit ribosomal RNA gene, partial sequence	2338	2338	100%	0.0	99.92%	OR003944.1
Penicillium oxalicum 18S ribosomal RNA (POX_rRNA045), rRNA	2338	2338	100%	0.0	99.92%	XR_007588136.1
Penicillium oxalicum 18S ribosomal RNA (POX_rRNA041), rRNA	2338	2338	100%	0.0	99.92%	XR_007588132.1
Penicillium oxalicum 18S ribosomal RNA (POX_rRNA039), rRNA	2338	2338	100%	0.0	99.92%	XR_007588130.1
Penicillium oxalicum strain I1R1 chromosome VIII	2338	13858	100%	0.0	99.92%	CP093060.1

The strain was identified as *Penicillium* and tentatively designated as *Penicillium oxalicum*.
